# Three decades of global ischemic heart disease burden: Trends, disparities, and drivers from 1990 to 2021

**DOI:** 10.1097/MD.0000000000050824

**Published:** 2026-09-25

**Authors:** Lianglei Hou, Yiwei Huang, Bin Lin, Anwu Huang

**Affiliations:** a Department of Cardiology, Wenzhou Central Hospital, Wenzhou, Zhejiang, China.

**Keywords:** disability-adjusted life-years, disease trends, Global Burden of Disease, ischemic heart disease, risk factors

## Abstract

Ischemic heart disease (IHD) remains a leading global health burden influenced by demographic transitions, socioeconomic disparities, and modifiable risk factors. Comprehensive analyses of global patterns are crucial to inform public health interventions. Using data from the Global Burden of Disease study 2021, we assessed global, regional, and national trends in IHD burden (1990–2021). Age-standardized rates of incidence, prevalence, mortality, and disability-adjusted life-years were evaluated. Trends were analyzed using estimated annual percentage change, age–period-cohort models, Bayesian forecasts, risk-factor attribution, decomposition analyses, health inequality, and frontier efficiency analyses. From 1990 to 2021, global IHD prevalence nearly doubled despite stable age-standardized rates, driven by population aging and growth. Eastern Europe exhibited consistently high disease burdens compared to sub-Saharan Africa. Significant gender disparities persisted, with higher burdens among males. High blood pressure, low-density lipoprotein cholesterol, and dietary risks dominated globally. Although age-standardized incidence and mortality declined, especially in high-sociodemographic index countries, absolute numbers increased due to demographic momentum. Forecasts suggest continued increases in absolute cases and deaths through 2035 despite declining standardized rates.Despite reductions in age-standardized IHD burden, substantial disparities persist. Future strategies should address demographic drivers, expand preventive efforts in lower-sociodemographic index regions, and enhance health system efficiency.

## 1. Introduction

Ischemic heart disease (IHD) remains a leading cause of global mortality and morbidity, accounting for a substantial portion of the noncommunicable disease burden worldwide. According to the Global Burden of Disease (GBD) 2021 study, IHD continues to be the primary cause of death globally, although mortality rates have declined in some high-income countries.^[1]^ Pathologically, IHD primarily arises from insufficient myocardial blood supply caused by coronary artery atherosclerosis, resulting in clinical presentations ranging from stable angina to acute coronary syndrome and ischemic cardiomyopathy.^[2]^

Despite advances in medical technology and therapeutic interventions, the global burden of IHD remains considerable. Particularly in low- and middle-income countries, the incidence and mortality rates of IHD continue to increase, driven by aging populations, unhealthy lifestyles, and rising prevalence of metabolic disorders.^[3]^ The World Health Organization’s 2023 Global Report on Cardiovascular Diseases highlighted that IHD accounts for over 30% of noncommunicable disease-related deaths globally.^[4]^

Epidemiologically, multiple risk factors influence the prevalence and mortality of IHD, including behavioral factors (such as smoking, unhealthy diet, and physical inactivity), metabolic conditions (including hypertension, diabetes, and hypercholesterolemia), and environmental exposures like air pollution and socioeconomic status.^[5]^ The GBD 2021 data reveals marked disparities in IHD burden across regions with varying sociodemographic index (SDI) levels: high-SDI regions have experienced declines in age-standardized mortality rates from IHD, whereas low-SDI regions have seen persistent increases in disease burden.^[6]^ These trends underscore substantial global challenges in IHD prevention and control, particularly in resource-limited settings. Recent GBD-based analyses have quantified global and regional IHD burden up to 2019 or focused on specific regions, but most have emphasized descriptive trends without integrating decomposition, inequality, and frontier analyses using the latest GBD 2021 estimates.^[7]^

Given these contexts, the present study aims to systematically evaluate the global, regional, and national disease burden of IHD using GBD data spanning 1990 to 2021, analyzing temporal trends, and attributing specific risk factors. This analysis will provide a scientific basis for targeted preventive and intervention strategies tailored to diverse regional contexts. Furthermore, the study emphasizes differences in disease patterns across age, sex, and SDI groups to elucidate key drivers of the global burden of IHD.

## 2. Materials and methods

### 2.1. Data sources and study design

This study utilized publicly available summary estimates from the GBD Study 2021 (GBD 2021), accessed through the Institute for Health Metrics and Evaluation GBD Results Tool in February 2025. Annual age-, sex-, and location-specific estimates for incidence, prevalence, mortality, disability-adjusted life-years (DALYs), years of life lost, and years lived with disability (YLDs) were extracted for 204 countries and territories from 1990 to 2021. All GBD estimates were generated using the DisMod-MR 2.1 Bayesian meta-regression framework, which enforces internal consistency across epidemiologic measures. Uncertainty was expressed as 95% uncertainty intervals (UIs), derived from 1000 posterior draws. No individual-level data were accessed.Because this study used only publicly available, de-identified aggregate GBD data and involved no individual-level participant information, ethics committee approval and informed consent were not required.

### 2.2. Disease definition

IHD was defined using International Classification of Diseases, 10th revision codes I20 to I25, including angina pectoris, acute myocardial infarction, and chronic IHD, following standardized GBD protocols.

### 2.3. Statistical analysis and modeling

#### 2.3.1. Burden estimation

Age-standardized incidence, prevalence, mortality, and DALY rates were computed per 100,000 population using the direct standardization method based on the GBD global standard population. DALYs were calculated as years of life lost + YLDs. Data were first imported in annual time-series format, cleaned, and stratified by sex, age group (5-year intervals), location, and SDI quintile.

#### 2.3.2. Temporal trend analysis

Trends were quantified using log-linear regression, with the estimated annual percentage change (EAPC) calculated as $(e^{\beta }-1)\times 100\%$, where β is the slope of the regression of the natural logarithm of the rate over time. Statistical significance was defined as a 95% UI not including 0. Joinpoint regression with permutation testing (*α* = 0.05) was used to detect statistically significant inflection points in long-term trends.

#### 2.3.3. Age–period–cohort modeling and forecasting

Nordpred annual percent change models were fitted using 5-year age and period intervals (1990–2021 baseline), with a power-5 link function and drift term restriction. Forecasts to 2035 were generated using Bayesian age–period–cohort (BAPC) models with random-walk priors for age, period, and cohort effects, implemented via integrated nested Laplace approximation. Posterior predictive distributions were summarized with median estimates and 95% credible intervals.

#### 2.3.4. SDI associations and decomposition

Linear regression quantified associations between SDI and age-standardized DALY rates. Population-attributable DALY changes were decomposed into population growth, population aging, and epidemiologic change using stepwise replacement decomposition.

#### 2.3.5. Risk-attributable burden

Population-attributable fractions were calculated as:


$$\begin{array}{c} PAF=\frac{\sum P(x)(RR(x)-1)}{\sum P(x)(RR(x)-1)+1} \end{array}$$

where P(x) denotes exposure distribution and RR(x) relative risk from meta-analyses. Nine modifiable risk factors were included, consistent with the GBD comparative risk assessment framework.

#### 2.3.6. Inequality and frontier analyses

Concentration curves and concentration indices were computed across SDI-ranked countries. Frontier analysis used a stochastic frontier approach to benchmark observed DALY rates against the lowest attainable rate at a given SDI level.

All countries and territories with available data were included in the analysis, while country labels in Figure 8 were limited to selected representative observations for visual clarity.

**Figure 1.**
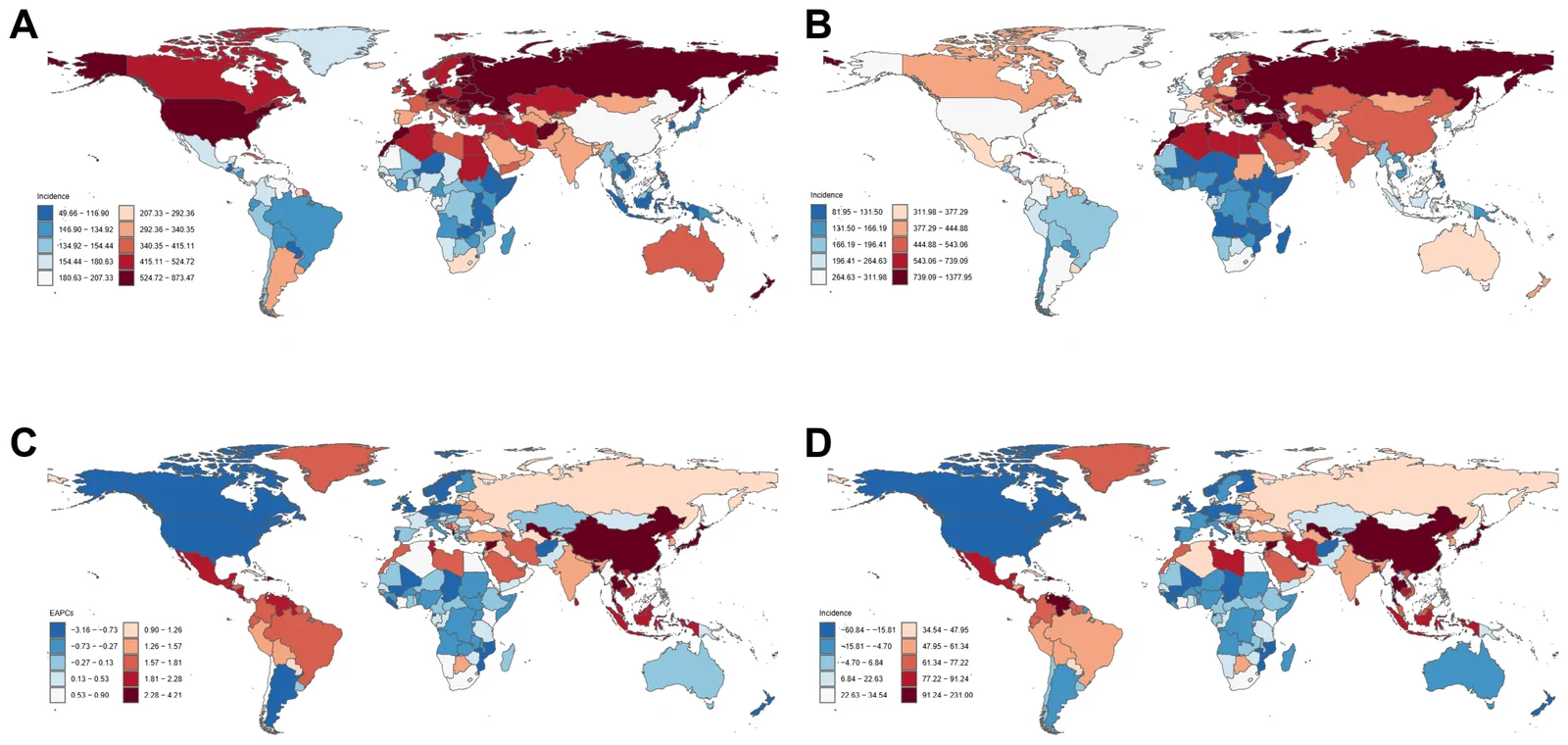
Geographic distribution and temporal trends of IHD incidence from 1990 to 2021. ASIR of IHD in (A) 1990, (B) 2021; (C) relative change (%) in ASIR between 1990 and 2021; (D) EAPC in ASIR from 1990 to 2021. Data are presented for 204 countries and territories based on GBD study 2021. ASIR = age-standardized incidence rate, EAPC = estimated annual percentage change, GBD = Global Burden of Disease, IHD = ischemic heart disease.

**Figure 2.**
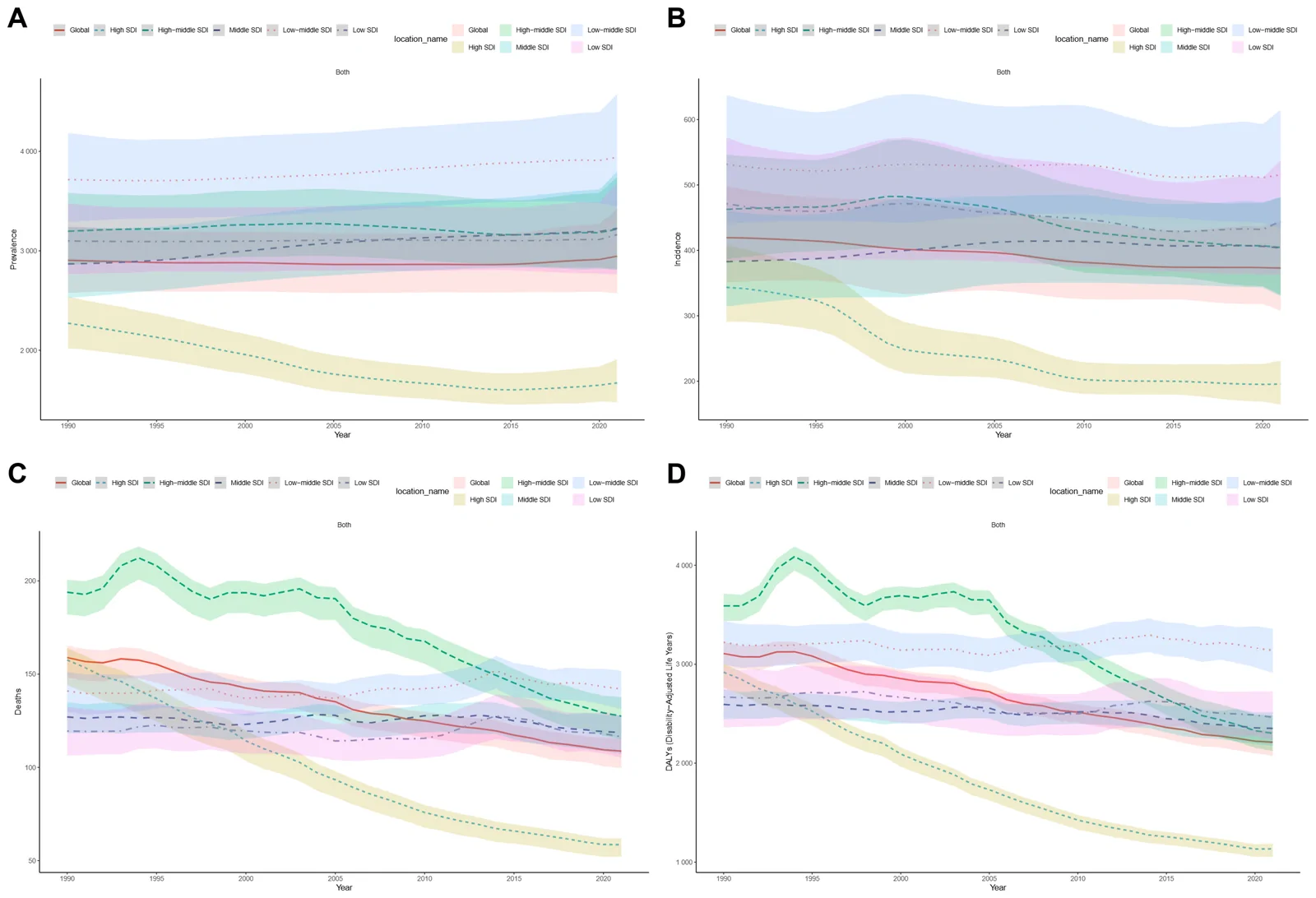
Temporal trends in age-standardized prevalence, incidence, death, and DALY rates of IHD by SDI quintiles globally, 1990 to 2021. (A) Age-standardized prevalence, (B) incidence, (C) deaths, (D) and DALYs per 100,000 population are shown across global SDI quintiles. Solid lines represent point estimates; shaded areas denote 95% UIs. DALY = disability-adjusted life-years, IHD = ischemic heart disease, SDI = sociodemographic index, UI = uncertainty interval.

**Figure 3.**
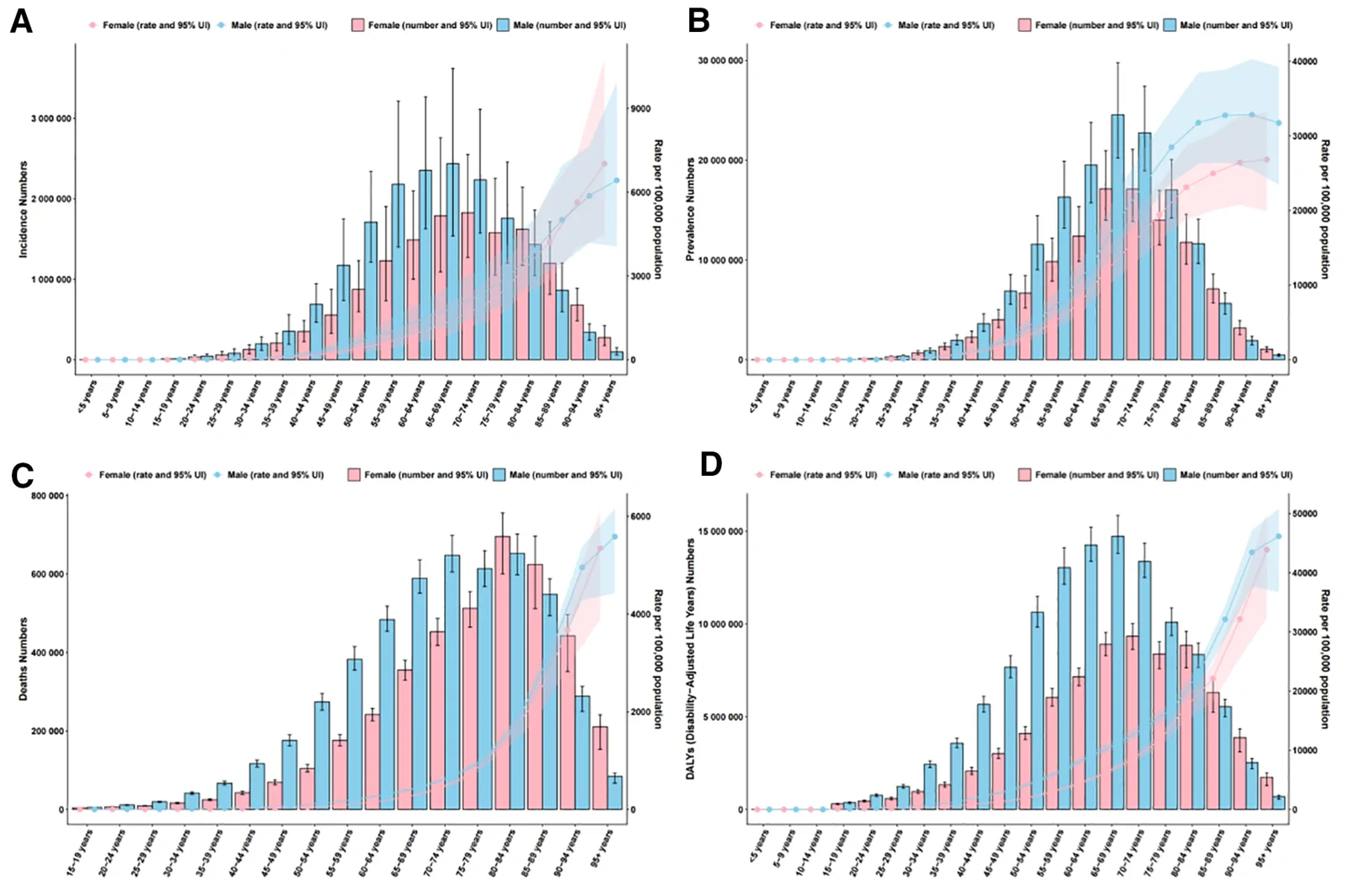
(A) Age- and sex-specific incidence, (B) prevalence, (C) deaths, (D) and DALYs of IHD globally in 2021. Bars represent absolute numbers, and lines represent age-specific rates per 100,000 population, stratified by sex and 5-year age groups. Error bars and shaded areas indicate 95% UIs. DALY = disability-adjusted life-years, IHD = ischemic heart disease, UI = uncertainty interval.

**Figure 4.**
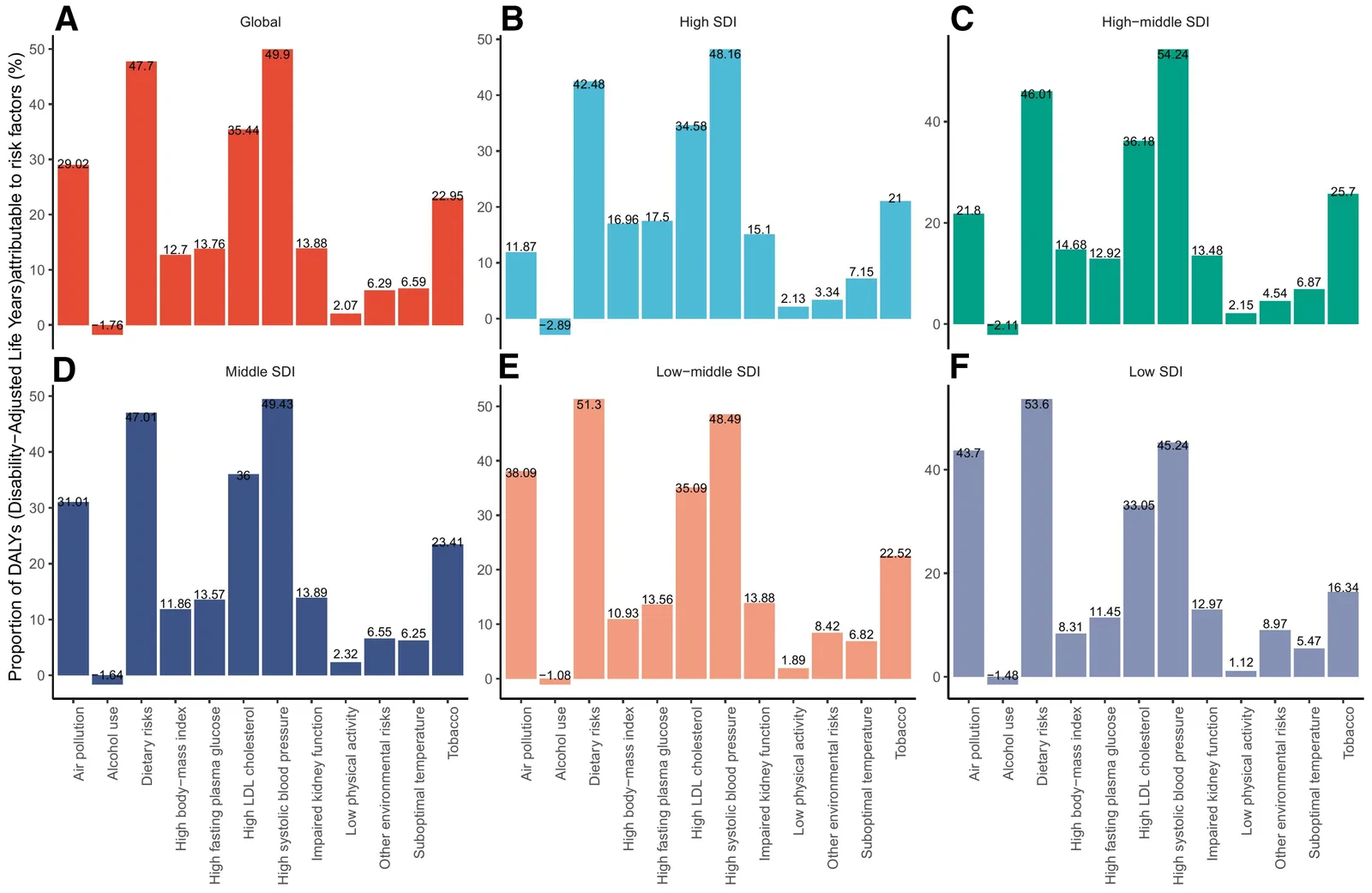
Proportion of IHD DALYs attributable to level 2 modifiable risk factors across global SDI strata in 2021.The stacked bar chart shows the percentage contributions of the top risk factors to IHD DALYs across 6 SDI strata. DALY = disability-adjusted life-years, IHD = ischemic heart disease, LDL = low-density cholesterol, SDI = sociodemographic index.

**Figure 5.**
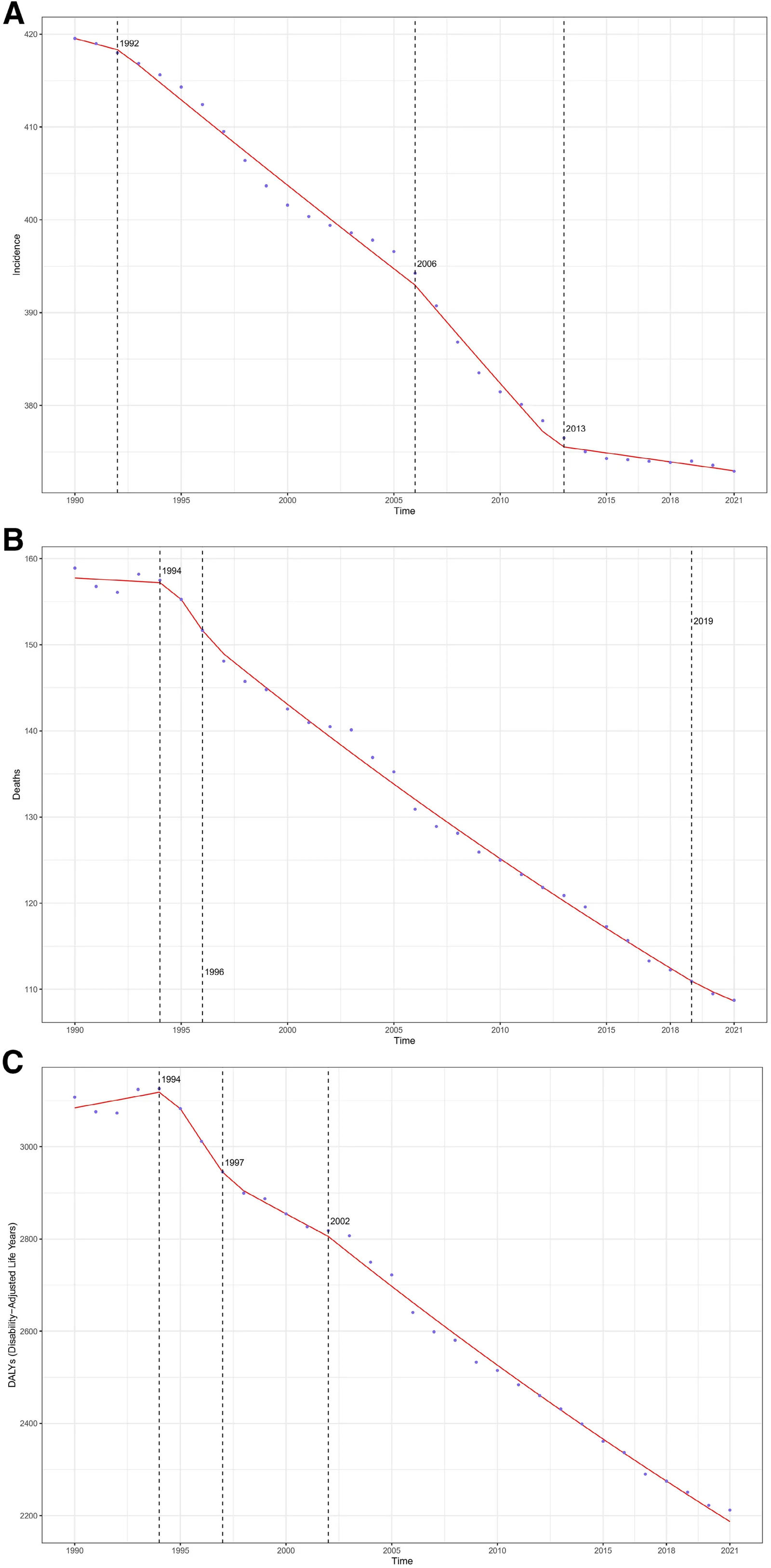
Temporal trends in the global burden of IHD from 1990 to 2021. (A) ASIR, (B) ASDR, and (C) age-standardized DALYs rate, each presented with AAPC. All rates are per 100,000 population. AAPC = average annual percentage change, ASDR = age-standardized death rate, ASIR = age-standardized incidence rate, DALY = disability-adjusted life-years, IHD = ischemic heart disease.

**Figure 6.**
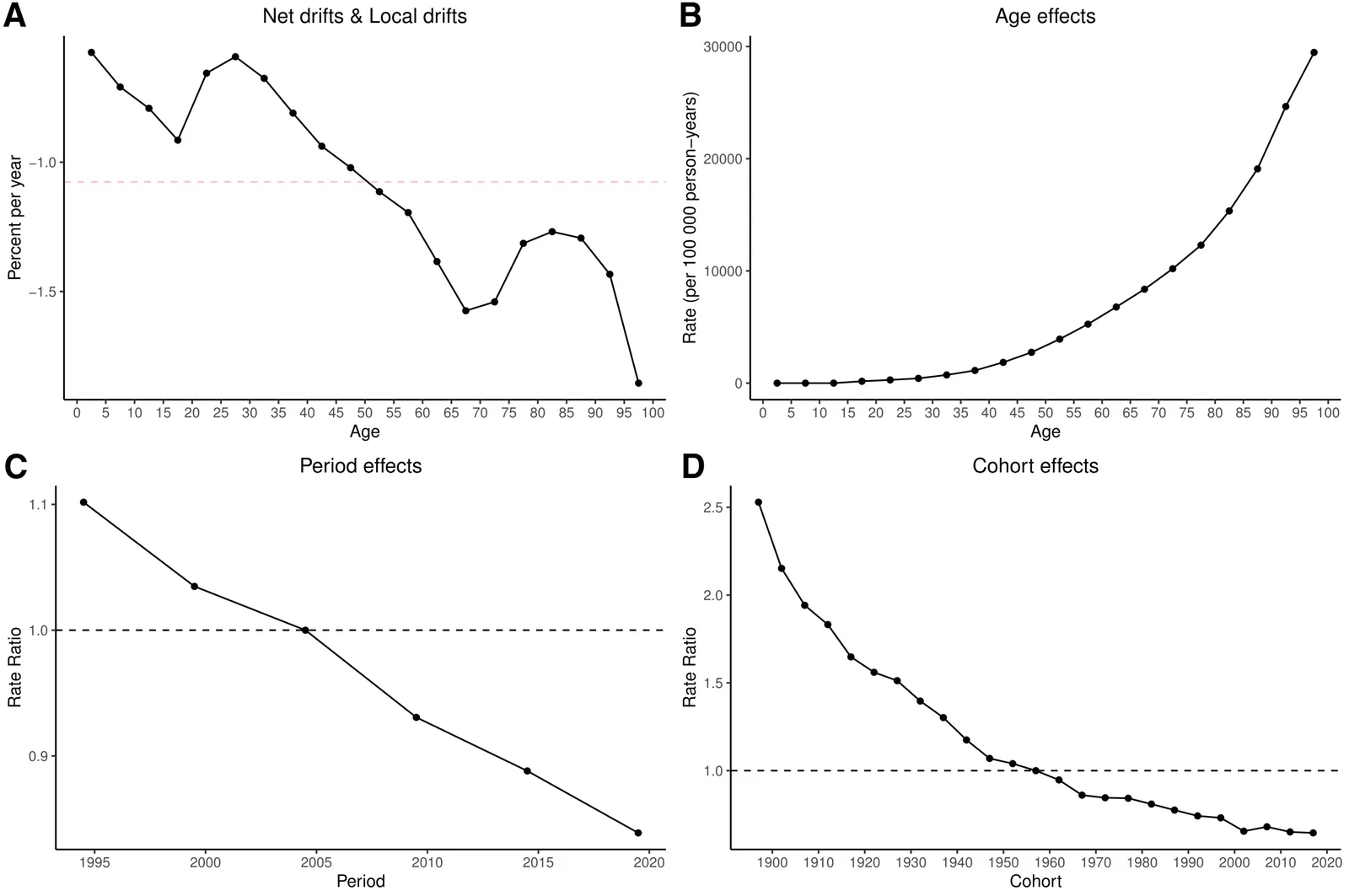
Net drift, local drifts, and age–period–cohort effects on IHD DALY rates, 1992 to 2021. (A) Net drift and local drift by age group (% per year); (B) Age effects (DALY rates per 100,000 person-years); (C) Period effects (rate ratios); (D) Cohort effects (rate ratios). DALY = disability-adjusted life-years, IHD = ischemic heart disease.

**Figure 7.**
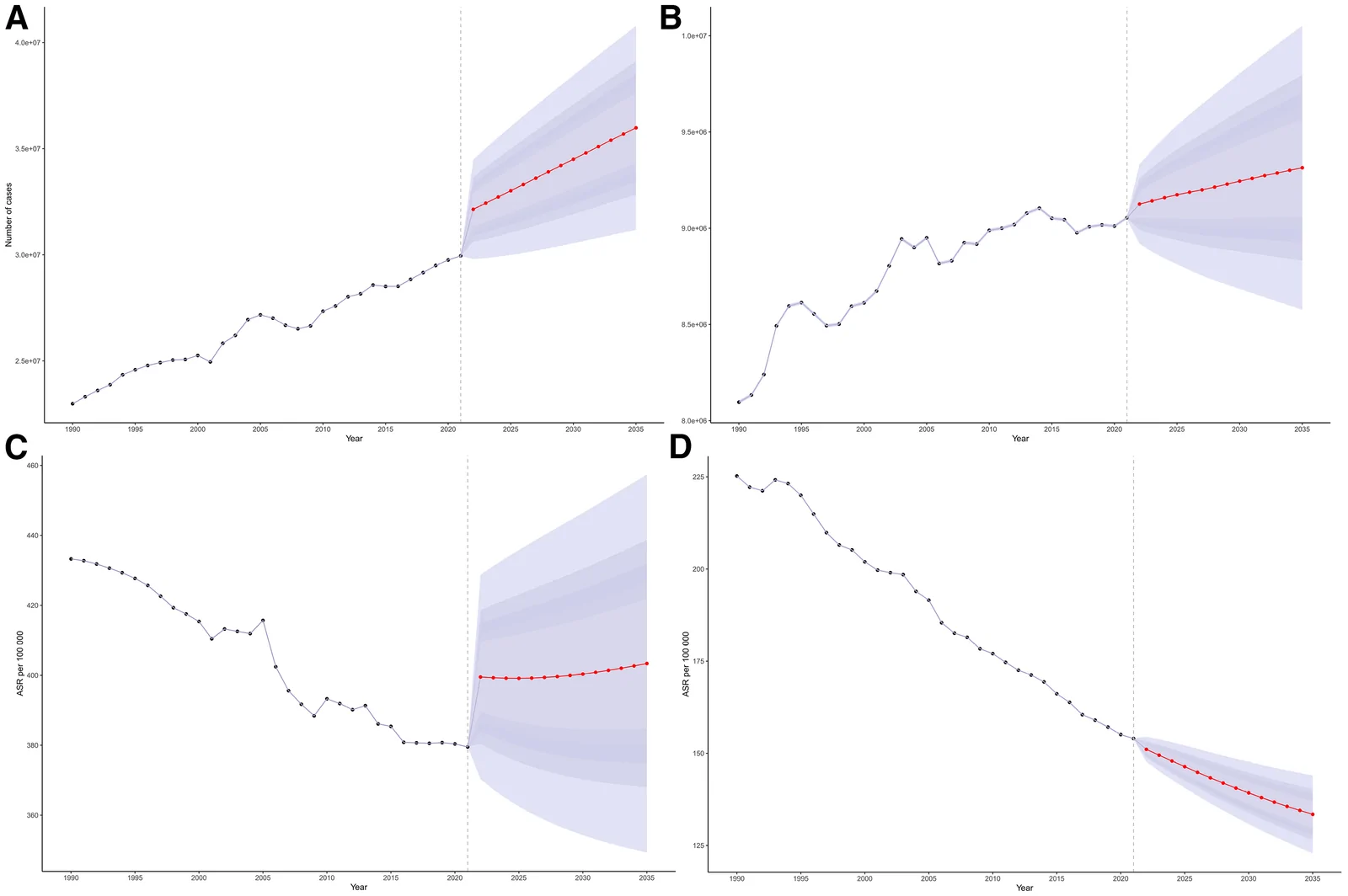
BAPC projections of IHD incidence and mortality from 2022 to 2035. The 4-panel chart presents BAPC-based projections of global incidence and mortality for IHD. (A) and (B) Projected crude numbers of global IHD incidence and deaths, respectively; (C) and (D) Corresponding age-standardized rates. Shaded areas indicate 95% UIs derived from the BAPC model. The forecasts suggest a continued increase in absolute case and death counts (A, B), alongside gradual declines in age-standardized incidence and mortality rates (C, D) through 2035. BAPC = Bayesian age–period–cohort, IHD = ischemic heart disease, UI = uncertainty interval.

**Figure 8.**
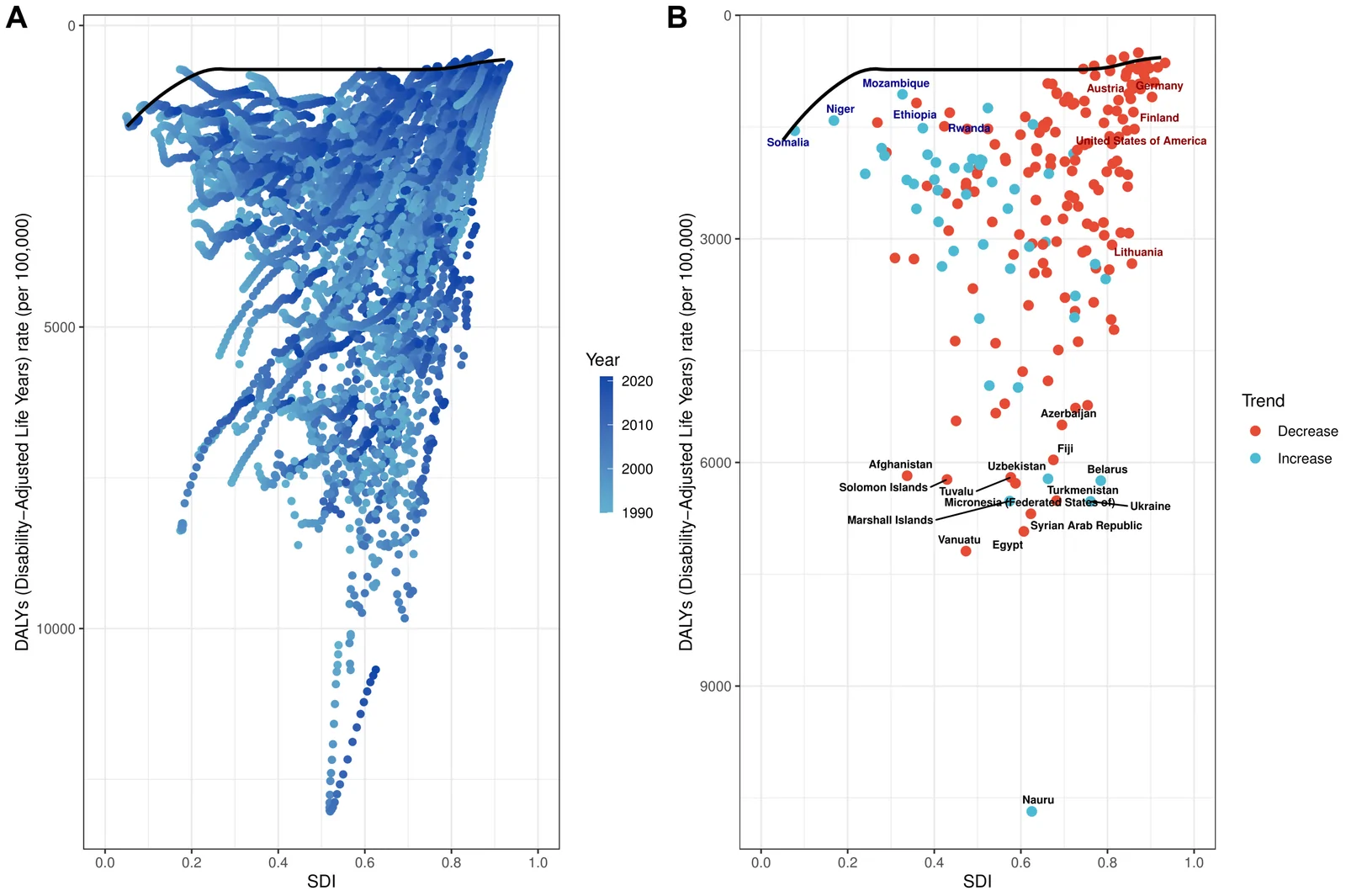
Frontier analysis of IHD DALY rates by SDI, 2021. The curve represents the lowest observed burden across SDI levels. Countries above the curve exhibit unrealized potential for improvement, while those near the frontier demonstrate higher efficiency in burden control. Each point represents a country or territory, and only selected representative countries are labeled for visual clarity. DALY = disability-adjusted life-years, IHD = ischemic heart disease, SDI = sociodemographic index.

#### 2.3.7. Software and reproducibility

All analyses were conducted in R (version 4.2.3; R Foundation for Statistical Computing). Key packages included mgcv, Epi, integrated nested Laplace approximation, nordpred, ggplot2, dplyr, and map. Data processing and visualization were supplemented by the JD_GBDR platform (version 2.36; Jingding Medical Technology Co., Ltd.), which standardized GBD data extraction and graphical rendering.

#### 2.3.8. Model validity and quality control

Model fit was assessed by inspecting residuals, comparing model-based estimates with observed GBD summary data, and confirming convergence for Bayesian components. Uncertainty was propagated by reporting 95% UIs for all estimates. Sensitivity checks confirmed consistency between EAPC-based trends and BAPC forecast trajectories.

## 3. Results

### 3.1. Global burden and prevalence trends of IHD (1990–2021)

This subsection draws on age-standardized prevalence estimates from GBD 2021, with temporal changes assessed using EAPC metrics. As described in Table 1, from 1990 to 2021, the global number of prevalent IHD cases increased substantially, from 282.9 million (95% UI: 214.9–361.6 million) to 597.0 million (95% UI: 457.3–752.9 million). Despite this striking increase in absolute numbers, the age-standardized prevalence rate (ASPR) slightly declined from 775.9 (95% UI: 592.4–990.8) per 100,000 in 1990 to 743.5 (95% UI: 571.2–938.3) in 2021. The EAPC was 0.01 (95% CI: −0.06–0.08), suggesting a largely stable global trend in prevalence after adjusting for population structure. This pattern implies that demographic shifts (particularly aging and population growth) rather than increases in per-person disease risk, were primary contributors to the rising burden. This interpretation is supported by the observed decline in the global age-standardized incidence rate (ASIR), which decreased from approximately 500 per 100,000 in 1990 to around 420 per 100,000 in 2021 (see Section 3.3).

**Table 1:** Prevalent cases and age-standardized prevalence rates of IHD by SDI level and sex, 1990 and 2021, and temporal trends from 1990 to 2021.

Characteristics	1990		2021		1990–2021	
	Prevalence cases No. ×10^5^ (95% UI)	ASPR per 100,000 (95% UI)	Prevalence cases No. ×10^5^ (95% UI)	ASPR per 100,000 (95% UI)	Incidence percentage change (95% CI)	EAPCs (95% CI)
Global	158.1 (131.8–188.5)	296.5 (247.1–353.4)	318.7 (262.8–382.7)	403.9 (333.1–484.9)	101.5 (98.9–104.4)	1.0 (0.9–1.0)
Male	90.7 (75.3–108.9)	337.7 (280.5–405.4)	179.6 (148.6–215.2)	453.6 (375.3–543.6)	98.0 (95.0–101.7)	2.3 (2.2–2.3)
Female	67.4 (55.9–80.8)	254.7 (211.2–305.0)	139.1 (114.5–166.8)	353.8 (291.1–424.2)	106.3 (102.1–110.4)	2.3 (2.2–2.3)
High SDI	37.7 (31.9–44.8)	428.4 (362.6–508.8)	39.9 (33.8–47.1)	364.7 (309.3–430.4)	5.9 (2.9–9.2)	−0.73 (−1.00–−0.46)
Middle SDI	36.5 (29.7–44.5)	212.1 (172.1–258.1)	105.0 (85.7–127.0)	428.8 (349.9–518.8)	187.3 (180.0–196.8)	2.4 (2.4–2.4)
Low SDI	10.0 (8.1–12.4)	200.3 (162.5–246.8)	21.9 (18.1–26.7)	196.1 (161.8–239.3)	118.1 (113.8–123.3)	−0.17 (−0.25–−0.10)
High-middle SDI	42.4 (35.4–50.4)	398.9 (333.1–473.6)	78.7 (64.6–94.5)	603.8 (495.2–724.6)	85.6 (80.5–90.4)	1.2 (1.1–1.3)
Low-middle SDI	31.3 (25.7–38.1)	269.1 (221.7–327.6)	72.9 (61.2–87.0)	379.6 (318.6–453.0)	133.4 (128.5–139.0)	1.2 (1.1–1.2)
Southeast Asia	5.5 (4.5–6.7)	118.6 (95.9–143.7)	14.2 (11.6–17.2)	203.2 (166.8–246.4)	157.1 (151.9–162.9)	1.8 (1.8–1.9)
Central Asia	2.8 (2.5–3.3)	406.9 (354.6–474.7)	5.7 (5.1–6.5)	598.1 (532.8–682.0)	103.2 (95.4–112.3)	1.2 (1.2–1.3)
East Asia	24.0 (19.4–29.1)	197.2 (159.4–239.2)	75.4 (60.1–92.3)	512.1 (408.1–626.7)	214.2 (200.3–232.0)	3.3 (3.2–3.4)
Western Europe	17.5 (15.8–19.7)	456.0 (409.9–513.3)	16.0 (13.8–18.5)	365.1 (316.3–423.7)	−8.90 (−13.56–−4.30)	−0.75 (−0.89–−0.62)
Oceania	0.1 (0.1–0.1)	144.4 (112.1–181.7)	0.3 (0.2–0.3)	181.3 (144.1–225.7)	167.0 (156.6–177.9)	0.8 (0.7–0.8)
Eastern Europe	18.4 (15.0–21.9)	810.3 (661.1–968.3)	24.9 (20.1–30.4)	1205.3 (971.7–1 –471.8)	35.8 (29.0–41.9)	1.1 (0.8–1.3)
High-income North America	15.8 (12.5–19.8)	562.0 (443.0–702.0)	11.1 (9.4–13.1)	301.1 (254.3–353.1)	−29.52 (−35.25–−21.94)	−2.46 (−2.80–−2.11)
Southern Latin America	1.4 (1.2–1.6)	282.0 (244.2–328.3)	1.7 (1.5–2.0)	257.8 (221.6–300.8)	24.9 (18.2–31.9)	−0.55 (−0.82–−0.29)
Central Europe	7.0 (6.1–8.1)	558.0 (486.9–643.7)	7.1 (6.1–8.1)	611.8 (531.6–705.5)	1.04 (−2.42–4.84)	−0.03 (−0.26–0.21)
Australasia	0.8 (0.7–1.0)	415.3 (362.9–479.7)	1.2 (1.0–1.4)	374.7 (308.4–451.0)	37.8 (26.8–48.9)	−0.40 (−0.66–−0.14)
High-income Asia Pacific	2.1 (1.7–2.5)	118.9 (95.9–145.0)	4.6 (3.7–5.8)	248.2 (197.7–311.5)	123.3 (100.1–147.9)	2.4 (2.2–2.6)
Caribbean	1.0 (0.9–1.2)	295.4 (246.6–348.0)	2.0 (1.6–2.3)	411.2 (340.1–488.3)	87.2 (81.9–92.9)	1.1 (0.9–1.2)
Andean Latin America	0.5 (0.4–0.6)	133.2 (109.0–161.4)	1.4 (1.1–1.7)	210.3 (171.7–256.5)	174.8 (165.7–183.9)	1.6 (1.5–1.7)
Tropical Latin America	1.8 (1.5–2.1)	118.3 (99.0–140.7)	4.3 (3.5–5.2)	189.6 (153.7–227.9)	138.9 (127.8–149.7)	1.8 (1.7–1.9)
Central Sub-Saharan Africa	0.8 (0.6–0.9)	140.3 (114.8–172.8)	1.8 (1.5–2.2)	132.6 (108.2–162.4)	135.5 (124.2–147.2)	−0.37 (−0.48–−0.27)
North Africa and Middle East	16.0 (13.8–18.9)	470.3 (407.5–556.4)	40.5 (35.0–47.9)	649.6 (562.5–768.1)	153.7 (145.7–161.6)	0.9 (0.8–1.1)
Central Latin America	2.8 (2.3–3.4)	170.4 (139.9–205.8)	7.8 (6.3–9.4)	306.5 (250.6–371.1)	176.9 (170.1–183.7)	1.9 (1.8–2.0)
South Asia	33.4 (26.7–41.2)	305.8 (244.2–376.8)	84.4 (68.5–102.2)	456.9 (371.1–553.6)	152.3 (145.9–159.5)	1.4 (1.3–1.4)
Southern Sub-Saharan Africa	1.1 (0.8–1.3)	202.9 (160.5–253.6)	2.1 (1.7–2.7)	265.5 (210.0–331.6)	100.5 (95.0–105.7)	0.7 (0.6–0.8)
Western Sub-Saharan Africa	3.1 (2.5–3.8)	160.4 (128.2–198.8)	7.2 (5.8–8.8)	146.6 (118.7–180.7)	131.7 (120.4–141.2)	−0.42 (−0.49–−0.36)
Eastern Sub-Saharan Africa	2.2 (1.8–2.8)	117.4 (93.1–146.6)	5.2 (4.1–6.4)	121.1 (97.3–150.8)	130.3 (122.8–137.7)	−0.02 (−0.17–0.12)

ASPR = age-standardized prevalence rate, CI = confidence interval, EAPC = estimated annual percentage change, IHD = ischemic heart disease, SDI = sociodemographic index, UI = uncertainty interval.

Substantial regional variation in IHD prevalence was observed. In 2021, Eastern Europe exhibited one of the highest ASPRs globally, exceeding 1100 per 100,000, while countries in sub-Saharan Africa generally reported much lower ASPRs, often under 400 per 100,000. Countries such as Pakistan and Egypt reported particularly high national-level ASPRs, while high-income countries like Japan and New Zealand remained among the lowest. These disparities reflect differences in diagnostic capacity, risk-factor exposures, and healthcare access across regions and SDI groups.

Significant gender differences were also evident. Males had a markedly higher ASPR in 2021 (935.5 [95% UI: 717.0–1183.8]) compared to females (561.0 [95% UI: 423.8–724.3]). Trends remained stable in both sexes: EAPC was −0.05 (95% CI: −0.12–0.03) for males and 0.06 (95% CI: −0.02–0.14) for females, indicating minimal sex-specific changes over the 3 decades.

### 3.2. Global and regional patterns of IHD incidence burden (1990–2021)

Analyses in this subsection were based on GBD 2021 age-standardized incidence data, visualized using geospatial mapping and evaluated through percentage change and EAPC analyses. Global maps of ASIR for IHD in 1990 and 2021 reveal persistent geographic disparities (Figs. 1A and 1B). In 1990, the highest ASIRs were observed in Eastern Europe and Central Asia, with rates exceeding 850 per 100,000 population in several countries. In contrast, sub-Saharan Africa, Southeast Asia, and parts of Latin America exhibited the lowest incidence rates, generally below 400 per 100,000. By 2021, the spatial pattern remained largely consistent, although a moderate increase in ASIR was observed across large parts of South Asia, North Africa, and Western sub-Saharan Africa. For example, Pakistan and Egypt exceeded 1000 per 100,000 in 2021, whereas countries such as Nigeria, Indonesia, and Peru maintained lower incidence rates below 500 per 100,000.

Relative changes in ASIR from 1990 to 2021 are shown in Figure 1C. Increases exceeding 100% were observed in several low- and middle-income countries, notably in South Asia and North Africa. Conversely, many high-income countries in Western Europe and Oceania saw reductions or minimal changes. These trends are further quantified in Figure 1D by EAPC, with the highest positive EAPCs (> 2.0%) observed in Pakistan, Uzbekistan, and Bangladesh. In contrast, countries like the United Kingdom, Canada, and Australia experienced modest declines (EAPC < –0.5), reflecting long-term success in cardiovascular prevention and care.

### 3.3. Temporal trends in incidence, prevalence, mortality, and DALYs of IHD, 1990 to 2021

Temporal trajectories across key indicators were evaluated using GBD 2021 age-standardized estimates and log-linear trend models, supplemented by Joinpoint regression to identify inflection points. From 1990 to 2021, the global burden of IHD demonstrated heterogeneous trends across key indicators (Figs. 2A–2D). Although the absolute number of prevalent cases steadily increased, the global ASPR remained relatively stable, fluctuating between 3000 and 3400 per 100,000 population. High-SDI regions consistently showed higher ASPRs than low-SDI regions, though this disparity narrowed slightly over time. In contrast, the ASIR declined moderately worldwide, from approximately 500 to 420 per 100,000, with the steepest reductions in high-SDI countries. Age-standardized death rates (ASDR) also fell substantially, particularly in high-SDI regions, while improvements in low-SDI areas were limited. A similar trend was observed for the age-standardized DALY rate, with faster declines in high-income regions and slower progress in low- and middle-SDI settings. These patterns collectively suggest global improvements in IHD burden under standardized metrics, yet substantial disparities remain across sociodemographic strata.

### 3.4. Age- and sex-specific distribution of IHD burden in 2021

Age- and sex-specific patterns were summarized using cross-sectional GBD 2021 estimates across standard 5-year age groups. In 2021, IHD showed a marked age-dependent increase across incidence, prevalence, mortality, and DALYs (Fig. 3). For the overall population, rates remained low before age 50 but rose sharply thereafter, peaking in the oldest age groups.For example, the age-specific prevalence rate increased from approximately 1173 per 100,000 at ages 40 to 44 to 27,901 per 100,000 at ages 85 to 89. Incidence followed a similar trajectory, increasing from approximately 581 per 100,000 at ages 50 to 54 to 4511 per 100,000 at ages 85 to 89.

Across younger and middle-aged groups, males generally had higher age-specific incidence, prevalence, mortality, and DALY rates than females, with the male–female gap becoming particularly evident after middle age. For example, at ages 60 to 64, the incidence rate was 1513.4 per 100,000 in males versus 905.1 in females, and among those aged 70 to 74, males had a death rate of 672.0 per 100,000 compared with 413.8 in females. At advanced ages, however, absolute burden showed a different pattern: from ages 80 to 84 onward, female numbers exceeded male numbers for incidence, prevalence, deaths, and DALYs. In contrast, male age-specific rates generally remained higher for prevalence, mortality, and DALYs even in the oldest age groups; only for incidence did the female rate exceed the male rate at ages 95+ years (7020.4 vs 6425.6 per 100,000). Thus, the late-life shift primarily reflected a greater absolute IHD burden among older women rather than a uniform reversal of age-specific rates.

### 3.5. Attributable burden of IHD by modifiable risk factors across SDI strata and regions in 2021

Risk-attributable burdens were quantified using the GBD comparative risk assessment framework, which estimates population-attributable fractions based on exposure distributions and established relative risks. In 2021, high systolic blood pressure was the leading contributor to IHD DALYs across all SDI strata, accounting for 48.16% of the attributable burden in high-SDI regions and 54.24% in high-middle-SDI regions (Fig. 4). High low-density lipoprotein cholesterol cholesterol (LDLC) and dietary risks ranked second and third, with their relative contributions varying by SDI level. Tobacco use, air pollution, and high fasting plasma glucose were also major contributors. As shown in Figure 4, alcohol use exhibited a negative attributable fraction globally (–1.8%) and in High-SDI regions (–2.9%), reflecting a modeled inverse association under the GBD’s nonlinear dose–response framework, discussed further below.

Across the 21 GBD regions, high systolic blood pressure was the leading contributor to IHD DALYs, with rates exceeding 2700 per 100,000 in Eastern Europe and 2300 in Central Asia. As shown in Figure S4, Supplemental Digital Content 1, dietary risks and high LDLC displayed similar regional distributions. Tobacco use contributed over 1100 DALYs per 100,000 in Eastern Europe, while air pollution, high body mass index, and kidney dysfunction played larger roles in South Asia and Sub-Saharan Africa.

Risk-attributable deaths closely paralleled DALY patterns (Fig. S4, Supplemental Digital Content 1), with alcohol use showing negative point estimates across all 21 GBD regions, although UIs overlapped the null in several regions.

### 3.6. Flattening progress in IHD burden: an average annual percent change (AAPC)-based analysis from 1990 to 2021

Temporal trends and inflection points were evaluated using Joinpoint regression, and long-term changes were summarized by calculating the AAPC. Long-term temporal trajectories were characterized using Joinpoint regression, from which AAPC values were derived. From 1990 to 2021, the global ASIR of IHD exhibited 2 distinct phases: a steady decline up until around 2013, followed by a pronounced flattening thereafter, with ASIR holding at approximately 410 (per 100,000) from 2013 through 2021 (AAPC before 2013 is approximately −1.8%, after 2013 is approximately −0.1%) (Fig. 5A). In contrast, the ASDR and age-standardized DALY rate continued to decline throughout the entire period without a clear plateau. Specifically, ASDR fell consistently from about 300 to 215 per 100,000 (overall AAPC −1.5%), and the DALY rate dropped from roughly 2800 to 2250 per 100,000 (overall AAPC −1.1%), both maintaining a steady downward trajectory even after 2015 (Figs. 5B and 5C).

The overall net drift in IHD DALYs was −1.06% per year from 1992 to 2021, reflecting a steady global decline (Fig. 6). Age-specific local drifts were negative across nearly all ages, with the sharpest reductions (over −1.2% per year) between ages 60 and 80. Period effects demonstrated a steady decrease in DALY risk over time, while cohort effects revealed a nearly 50% lower burden in individuals born after 1980 compared to those born before 1920.

### 3.7. Forecasted trends in IHD incidence and mortality through 2035

Future incidence and mortality were projected using a BAPC model, incorporating random-walk priors for age and period effects. Between 1990 and 2021, IHD cases rose steadily, reaching 36.4 million in 2021. BAPC projections indicate that this figure will exceed 41.2 million by 2035, with the trajectory of absolute case numbers becoming steeper in the latter part of the forecast horizon (Fig. 7A). Despite the growing absolute burden, the global ASIR declined from 480.3 to 419.6 per 100,000 between 1990 and 2021 and is expected to drop further to 387.4 per 100,000 by 2035.

A comparable pattern was observed for mortality. IHD deaths are projected to rise from 9.04 million in 2021 to 10.12 million by 2035, with a more pronounced increase in the absolute number of deaths towards the end of the projection period (Fig. 7C). In contrast, the ASDR is anticipated to decline from 214.9 to 194.7 per 100,000 over the same period, indicating that the projected steep rise in case and death counts is driven primarily by demographic factors rather than an abrupt worsening of age-standardized risk.

### 3.8. Efficiency gaps in IHD burden reduction: frontier analysis across SDI levels

Efficiency gaps were examined using frontier analysis, which evaluates each country’s age-standardized DALY rate relative to the optimal performance achievable at its SDI level. Substantial variation existed among countries with comparable SDI levels in 2021 (Fig. 8). Several high-SDI nations (such as the United States, Germany, and Poland) lay well above the frontier curve, with age-standardized DALY rates exceeding 3000 per 100,000 despite SDI values above 0.85. In contrast, countries like Ethiopia, Rwanda, and Vietnam were positioned much closer to the frontier, with DALY rates of 1500 to 1800 per 100,000, despite SDIs as low as 0.45 to 0.65.

The wide vertical deviations (over 2000 per 100,000 in some high-SDI countries) highlight major disparities in how efficiently nations convert sociodemographic development into reduced IHD burden. The frontier curve (Fig. 8) serves as a benchmark for optimal performance at each SDI level, offering a comparative framework to identify underperforming countries and guide targeted improvements.

### 3.9. Supplementary analyses on sociodemographic and temporal patterns

We additionally performed several supplementary analyses (Figs S2–S7, Supplemental Digital Content 2) to explore disparities and long-term patterns in IHD burden. Specifically, these supplementary assessments incorporated concentration indices and concentration curves to quantify inequality, age–period–cohort modeling to characterize generational trends, and demographic decomposition to distinguish epidemiologic from population-driven changes. These analyses revealed persistent sex differences, widening inequality across SDI levels, and heterogeneous progress among regions. A strong inverse relationship between SDI and DALY rates was evident, and countries with higher baseline burdens or lower development levels tended to experience slower improvements. Age–period–cohort modeling showed generational gains but plateauing recent trends. Decomposition analysis highlighted the dominant roles of population aging and growth in shaping DALY increases, while concentration curves and regression results confirmed an expanding global inequality in IHD burden.

## 4. Discussion

This comprehensive analysis of the global IHD burden from 1990 to 2021 reveals both notable achievements and persistent challenges. Although age-standardized rates of prevalence (ASPR), incidence (ASIR), mortality (age-standardized mortality rate), and DALYs have declined in many high-SDI regions (reflecting improvements in cardiovascular care and prevention) the absolute number of IHD cases has more than doubled. This paradox is largely driven by population aging and growth, underscoring how demographic transitions are reshaping the global chronic disease landscape and challenging healthcare systems. Our finding of declining age-standardized rates but rising absolute numbers of IHD cases and deaths is consistent with prior GBD-based cardiovascular analyses, which have similarly attributed the growing absolute burden to population aging and growth rather than worsening per-person risk.^[3,7]^

The distribution of IHD burden remains highly unequal across geography, sex, and socioeconomic status. Eastern Europe continues to experience disproportionately high prevalence, associated with entrenched risk factors such as tobacco use, dietary risks, and hypertension, and exacerbated by limited improvements in healthcare infrastructure. In contrast, low reported burdens in sub-Saharan Africa may reflect not only younger populations but also substantial underdiagnosis due to limited diagnostic access. Incidence trends further highlight regional disparities: while high-income countries have achieved modest declines through effective screening and management, South and Central Asia, North Africa, and parts of the Middle East have seen sharp increases, driven by urbanization, demographic shifts, and inadequate control of modifiable risks.

Notably, gender disparities persist, with males bearing higher incidence and DALY burdens than females across younger and middle-aged groups in most regions. However, these gaps are narrowing in high-SDI countries, possibly due to improved cardiovascular management in men and rising exposure to smoking and obesity in women. In contrast, the gender gap remains wide in low- and middle-SDI regions, driven by unequal access to care and enduring sociocultural barriers (Fig. S4, Supplemental Digital Content 1). At advanced ages, the sex pattern became more complex. Although males generally retained higher age-specific IHD rates, women accounted for a greater absolute burden from approximately 80 years of age onward. Selective survival may partly explain this pattern: higher premature IHD mortality and competing risks among men may reduce the number of susceptible male survivors reaching very old age, whereas women generally live longer and therefore constitute a larger proportion of the oldest population. In addition, biological and clinical factors, including postmenopausal changes, cumulative cardiometabolic risk exposure, and sex differences in the recognition and treatment of IHD, may contribute to the narrowing sex gap at advanced ages.^[8]^ These mechanisms cannot be distinguished using aggregate GBD data and should therefore be considered complementary explanations rather than definitive causal mechanisms.These patterns call for sex- and region-specific prevention strategies that adapt to evolving exposures and health system capabilities.

Our trend analyses add further nuance. While ASPR remained relatively stable, the cumulative burden rose due to increased life expectancy and disease duration. Age-specific analyses revealed that DALY rates rose sharply after age 60, reaching over 55,000 per 100,000 in those aged 85 to 89. Period and cohort effects show a 25% reduction in DALY risk since the 1990s and substantial generational improvements, especially for those born after 1950. However, the recent flattening of these trends suggests that the benefits of current prevention strategies may be plateauing, prompting a need to address emerging challenges like obesity, diabetes, and prolonged survival with residual disability. This emerging plateau in IHD improvements is consistent with reports from several high-income countries, where rising obesity, diabetes, and persistent social inequalities have begun to slow earlier declines in CVD mortality.^[9]^

Advanced modeling using a BAPC framework further contextualizes these patterns. BAPC forecasts indicate that, despite continued declines in age-standardized incidence and mortality, the absolute numbers of IHD cases and deaths will keep rising through 2035, with a visibly steeper increase in the latter part of the projection horizon, particularly in middle- and low-SDI settings (Figs. 7A–7C). This apparent sharp rise is driven largely by demographic forces (rapid population aging and overall population growth, together with expanding diagnostic capacity) rather than an abrupt worsening of age-standardized risk. At the same time, these projections rest on the assumption that recent age–period–cohort patterns persist into the future and rely on GBD estimates for older age groups, where data quality and cause-of-death attribution are more uncertain; they should therefore be interpreted with caution, especially for mortality at the highest ages, as indicative of the direction and relative magnitude of change rather than precise point estimates. Even under these conservative assumptions, the forecasts highlight the urgency of strengthening surveillance and adapting health system responses to an expanding IHD burden in aging populations. DALY-based BAPC forecasts were not presented because validated models for disability outcomes remain limited; future studies should extend forecasting frameworks to YLDs as more robust disability data and forecasting tools become available. Similar BAPC-based projections in national and regional IHD studies have also suggested that demographic momentum will sustain increases in absolute IHD events over the coming decades, even under scenarios of modest improvements in age-specific rates.^[10]^

Supplementary analyses of sociodemographic and temporal patterns further supported these findings. Decomposition indicated that, in most low- and middle-SDI regions, the majority of the increase in IHD DALYs was driven by population aging and overall population growth rather than worsening age-specific rates, while concentration-curve analyses showed that IHD DALYs have become increasingly concentrated in low-SDI countries over time. In addition, a series of SDI- and region-stratified plots provided more granular views of age–sex patterns, inequality metrics, and robustness checks for the main results (Figs. S1–S7, Supplemental Digital Content 3).

Frontier analysis provides further insight into performance variability. Even among countries with similar SDI levels, health system efficiency varies markedly. For instance, the United States and Germany exhibit much higher DALY rates than expected for their development level, whereas countries like Vietnam and Rwanda perform close to the efficiency frontier. These patterns indicate that improving IHD outcomes is not solely a matter of socioeconomic development but also of how effectively health systems translate resources into prevention, primary care, and acute cardiovascular care, a finding consistent with prior work showing large CVD mortality gaps between countries with comparable resources but differing health system performance.^[3]^ Benchmarking against top performers could therefore support strategic improvement, particularly in underperforming high-SDI countries.

The global risk profile of IHD remains dominated by metabolic risk factors—especially high systolic blood pressure and LDLC, accounting for the majority of attributable DALYs. However, their relative importance varies by region: dietary risks dominate in upper-middle-SDI settings, while LDLC is more influential in high-SDI regions. Air pollution, tobacco use, and elevated fasting glucose are also prominent contributors in many regions. These differences underscore the importance of tailoring interventions to local epidemiologic and resource contexts.

A particularly notable and controversial result is the negative population-attributable fraction for alcohol use in certain regions, especially those with high SDI. This does not imply that alcohol is universally protective, but rather reflects a modeling construct in which low-to-moderate intake (commonly observed in high-income populations) is associated with lower IHD risk compared to abstinence or heavy drinking. This J-shaped association has been widely described, including in reviews by O’Keefe et al (2014) and pooled analyses by Wood et al (2018), which reported lower IHD risk and mortality among moderate drinkers.^[11,12]^

However, these findings remain under intense debate. A large 2023 cohort study published in Nature Medicine found no safe threshold for alcohol consumption, with increased risk of over 60 diseases, including cardiovascular conditions, even at low levels.^[13]^ Such contradictions suggest potential residual confounding, reverse causality, or selection bias in earlier observations.^[14]^ Therefore, the negative alcohol-attributable fraction should be interpreted as a model-dependent estimate rather than evidence of a causal cardioprotective effect or a clinical recommendation for alcohol consumption.

More broadly, although GBD 2021 applies a standardized estimation framework across locations, the availability and quality of underlying data remain heterogeneous. In low-SDI settings, limited diagnostic capacity, incomplete vital registration, and sparse epidemiological surveillance may contribute to underestimation of IHD burden. Changes in diagnostic capacity and surveillance over time may also influence apparent temporal trends, as improved case ascertainment can increase estimated burden independently of true changes in disease occurrence. Therefore, both regional burden estimates and apparent proximity to the frontier in data-sparse settings should be interpreted cautiously.

Taken together, these global and regional patterns support a dual strategy: maintain universal priorities like hypertension and lipid management, while tailoring regional approaches, reducing tobacco and alcohol in Eastern Europe, addressing metabolic risks in South Asia, and mitigating air pollution in sub-Saharan Africa. In particular, alcohol-related findings should be interpreted cautiously and incorporated into broader, context-sensitive public health messaging.

Rather than concluding with a generic summary, the findings from this study demand sustained, equity-driven public health investment. The complex interplay between aging, socioeconomic conditions, health infrastructure, and emerging risk patterns calls for a shift from one-size-fits-all strategies to more adaptive, locally responsive interventions. Bridging global disparities in IHD burden will require both technical excellence and policy-level resolve.

## 5. Conclusion

This study offers a comprehensive assessment of the global IHD burden from 1990 to 2021, revealing meaningful progress in reducing age-standardized rates, alongside persistent challenges driven by demographic aging, widening socioeconomic disparities, and variable health system effectiveness. While standardized rates have declined, absolute numbers of cases and deaths continue to rise—particularly in middle- and low-SDI regions, where epidemiological gains remain limited. Achieving equitable reductions in IHD burden will require closing infrastructure gaps, expanding preventive measures, and enhancing the efficiency of health resource allocation worldwide.

## Acknowledgments

This research was funded by the Zhejiang Province Medical and Health Science and Technology Planning Project (grant No. 2023KY1154). The authors declare no competing interests.

## Author contributions

**Conceptualization:** Anwu Huang.

**Data curation:** Lianglei Hou.

**Formal analysis:** Yiwei Huang.

**Methodology:** Anwu Huang.

**Visualization:** Bin Lin.

**Writing – original draft:** Bin Lin, Anwu Huang.

**Writing – review & editing:** Lianglei Hou.














